# LncRNA NONMMUT055714 acts as the sponge of microRNA-7684-5p to protect against postoperative cognitive dysfunction

**DOI:** 10.18632/aging.202932

**Published:** 2021-04-26

**Authors:** Changwei Wei, Yi Sun, Jing Wang, Dandan Lin, Victoria Cui, Hui Shi, Anshi Wu

**Affiliations:** 1 Department of Anesthesiology, Beijing Chao-Yang Hospital, Capital Medical University, Beijing, China; 2 Department of General Surgery, MedStar Georgetown University Hospital, Washington, D.C., USA; 3 Department of Clinical Psychology, Beijing Chao-Yang Hospital, Capital Medical University, Beijing, China

**Keywords:** LncRNA NONMMUT055714, POCD, MiR-76845p, SORLA, Aβ deposition

## Abstract

Postoperative cognitive dysfunction (POCD) is a neurological complication of surgery especially common in elderly patients. In this study, we investigated the role of NONMMUT055714 in POCD via regulation of miR-7684-5p. In a POCD mouse model, we induced overexpression of NONMUTT055714 via transfection of lentivrus into the hippocampus, and used the Morris water maze for assessment of cognitive function. Silencing of NONMUTT055714 and miR-7684-5p was induced in primary hippocampal neurons to observe the effects of these regulatory RNAs on cellular processes. Bioinformatics analysis and a double luciferase reporter experiment were performed to further explore the relationship between NONMMUT055714, miR-7684-5p, and SORLA. Cell and animal rescue experiments were performed to verify the ability of miR-7684-5p to reverse the protective effects of NONMMUT055714 overexpression in POCD. We observed that NONMMUT055714 has decreased expression in the POCD mouse model. Overexpression of NONMMUT055714 protected against cognitive impairment of the POCD mouse model *in vivo*. We identified miR-7684-5p as a NONMMUT055714-related miRNA and in turn as an upstream regulator of SORLA. We found that NONMMUT055714 downregulation is associated with decreased SORLA, increased Aβ and p-tau expression, increased inflammatory biomarkers, increased markers of oxidative stress, and increased neuronal apoptosis *in vitro*. The effects of NONMMUT055714 downregulation were reversed by silencing miR-7684-5p *in vitro* and *in vivo*. Taken together, our findings suggest that NONMMUT055714 is protective against the development of POCD via its function as a ceRNA (or miRNA sponge) in the regulation of miR-7684-5p and SORLA. We therefore propose NONMMUT055714 as a novel target for the investigation and prevention of POCD.

## INTRODUCTION

Postoperative cognitive dysfunction (POCD) is a common neurological complication following surgery, especially in elderly patients [1]. While varied in presentation and timing, the main clinical manifestations are disorders in memory, orientation, attention, perception, and consciousness, which can last for weeks or even months [2].

POCD is associated with higher incidence of postoperative complications, delayed recovery, reduced quality of life, and increased mortality [3]. A previous study involving 644 patients found that 17% of patients with total hip joint replacement surgery and 43% of patients with coronary artery bypass graft surgery developed POCD at 7 days postoperatively, and incidence of POCD in both groups remained at 16% three months later [4]. Increased age, major surgery, history of myocardial infarction, history of alcohol dependence, and pre-existing cognitive impairment have been identified as independent risk factors for POCD [5–7]. While the details of POCD pathogenesis remain unclear, it is thought that inflammatory responses, oxidative stress, changes in protein expression, apoptosis, and degeneration of the central cholinergic system may be contributing factors [8, 9]. POCD may even share a common pathogenesis with Alzheimer’s disease, which is associated with the deposition of amyloid beta (Aβ) and hyperphosphorylation of tau protein in the hippocampus [10]. In a mouse model, isoflurane anesthesia was shown to induce high levels of Aβ peptide formation, suggesting a possible mechanism for postoperative exacerbation of Alzheimer’s disease and associated cognitive decline [11]. There is an urgent need to understand the underlying mechanisms leading to POCD and identify effective methods to protect cognitive function after surgery.

Long non-coding RNAs (lncRNAs) are RNA molecules greater than 200 nucleotides in length which participate in regulation of gene expression [12]. In the brain, lncRNAs exhibit temporally- and spatially-specific patterns of expression relevant to the development and neurological function of the central nervous system [13]. LncRNA regulatory networks appear to correlate with behavior and memory formation, and their dysfunction may be involved in the development of neurological disorders and neurodegeneration [14]. LncRNAs have been hypothesized to act as competing endogenous RNAs (ceRNAs), alternatively known as natural microRNA sponges, by binding to and regulating microRNAs (miRNAs) [15, 16]. In turn, miRNAs, which are short non-coding RNAs, act as important post-transcription regulators of gene expression.

Previous microarray analysis performed by our group demonstrated that the lncRNA NONMMUT055714 is significantly downregulated in the hippocampus of a POCD mouse model compared to control mice [17]. We suggested based on a constructed ceRNA network that NONMMUT055714 competitively binds to the miRNA miR-7684-5p, regulating the expression of sorting-related receptor with A-type repeats (SORLA, also known as LR11 or SORL1) [17]. Defects in SORLA have been implicated in the pathogenesis of Alzheimer’s disease due to the protein’s role as an intracellular sorting receptor, including for amyloid precursor protein (APP), the precursor to Aβ [18, 19].

In this study, we hypothesize that decrease of expression NONMMUT055714 contributes to the development of POCD via regulation of miR-7684-5p. First, we evaluate the expression of NONMMUT055714 in a POCD mice model. Second, we explore the effects of NONMMUT055714 on neurotoxic protein level, tau phosphorylation, inflammatory response, oxidative stress, and neuronal apoptosis in primary hippocampal neurons. Next, we test whether miR-7684-5p is in turn involved in NONMMUT055714 regulation. Finally, we investigate the effects of silencing and overexpression of NONMMUT055714 *in vitro* and *in vivo*. Our study aims to elucidate and define NONMMUT055714-driven molecular mechanisms in the development of POCD, providing rationale for NONMMUT055714 as a novel therapeutic target for POCD prevention.

## MATERIALS AND METHODS

### Animals

C57BL/6 elderly male mice (12-14 months) weighing 25-35g were obtained from Beijing Vital River Laboratory Animal Technologies (Beijing, China). The mice were initially kept in cages for one week so that they could adapt to the environment. Animals received food and water *ad libitum* with a constant room temperature of 23-25° C and a relative humidity of 50–60%. The room was well-ventilated, with a 12 hour light/dark cycle each day. These experiments were conducted strictly in accordance with guidelines for the care and use of animals and were approved by the hospital animal ethics committee at Capital Medical University (NO: AEEI-2020-117).

### Establishment of the POCD mice model

Mice received orthopedic surgery as we reported [17] and Terrando et al. previously described [20]. The surgical procedure was performed under 2% isoflurane general anesthesia. A longitudinal incision was made on the left hind paw of each mouse. A 0.38 mm pin was then inserted in the tibial medullary canal, followed by performance of an osteotomy. The skin was closed with 5-0 nylon sutures. 0.2% ropivacaine was injected subcutaneously for postoperative analgesia. Isoflurane was discontinued immediately after the operation, and animals were placed back in their cages, where they awoke naturally. During the procedure, temperature was maintained at 37° C using a heating pad.

### Grouping and stereotaxic injection

Mice were randomly divided into six groups: sham, POCD, NONMMUT055714 (055714) + POCD, negative control (NC) + POCD, miR-7684-5p + 055714 + POCD, and miR-7684-5p inhibitor + 055714 + POCD. Sham and POCD group mice did not receive viral vectors. All mice besides the sham group received tibial fracture surgery with intramedullary pinning as described above. For all other groups, mice were anesthetized with Avertin (tribromoethanol) and head-fixed on a stereotaxic apparatus. An incision was made on the skull. 055714-overexpressed/NC lentivirus and/or miR-7684-5p mimics/inhibitor lentivirus in a total volume of 2 μl was injected bilaterally into the hippocampal CA1 area (coordinates: -2.0 mm anteroposterior, ±1.5 mm mediolateral, -1.5 mm dorsoventral from the bregma). After stereotaxic injection, the incision was closed. Mice were allowed to recover and returned to their cages. Behavioral training testing was performed 2 weeks following treatment with lentivirus.

### Morris water maze

The Morris water maze test was selected to assess spatial learning and memory [21]. The classic Morris water maze test includes a water maze navigation test and probe trail test. In the water maze navigation test, water was added into the tank to about 30 cm height, and the platform was placed in the first quadrant less than 1 cm under the surface of the water. A small amount of titanium dioxide powder was sprinkled into the water, which was stirred and mixed so that the platform was not visible. The water temperature was kept at 23 ± 2° C. At the beginning of the experiment, mice were placed on the platform for 30 seconds to promote familiarity with the testing environment. The mice were then placed into the water with their heads facing toward the wall of the pool at four quadrants in turn, and the time that they took to find the hidden platform was recorded. If the time required exceeded 60s, the animal was guided to the platform and allowed to stay on top of the platform for 30s. This training was repeated four times in a fixed period every day for 6 consecutive days before surgery with the aim of training mice to find the underwater platform. The probe trail test was performed on the third day after surgery. The submerged platform was removed. The mice were lowered headfirst into the water of the contralateral quadrant of the original platform. The percentage of time spent in the target quadrant, latency, and swimming speed of were recorded.

### Cell culture and transfection

Primary hippocampal neurons were cultured from postnatal day 0 (P0) C57BL/6 mice. Bilateral hippocampi were dissected, and the hippocampal tissue was washed twice with PBS and digested with 0.25% trypsin (Gibco, CA, USA) for 10 minutes. An equal volume of DMEM-F12 medium (Gibco, CA, USA) with 10% fetal bovine serum (FBS) (Gibco, CA, USA) was added to stop digestion. The supernatant was collected and centrifuged at 1000 rpm for 5 minutes, the resulting supernatant was discarded, and implant solution (DMEM-F12 medium with 10% FBS) was added. The cell suspension was dripped onto a cover slip coated with poly-D-lysine (Invitrogen, CA, USA) and incubated at 37° C. After half of an hour, neurobasal medium (Gibco, CA, USA) with Pen-Strep (Invitrogen, CA, USA), B27 (Gibco, CA, USA) and GlutaMAX (Thermo Fisher, MA, USA) was added. Si-055714, si-NC, miR-7684-5p inhibitor and miR-NC were obtained from Generalbiol (Chuzhou, China). Neurons were transfected with Lipofectamine™ 2000 (Invitrogen, CA, USA) on DIV4.

### Prediction of molecular targets and dual-luciferase reporter assay

The potential targets of NONMMUT055714 were predicted using the bioinformatics databases MiRanda and PicTar. Potential binding targets of miR-7684-5p were identified using the bioinformatics algorithm TargetScan. To further explore the underlying mechanism of the lncRNA/miRNA regulatory function of NONMUT055714 and miR-7684-5p, dual-luciferase reporter assay was performed. The putative miR-7684-5p binding sequences of the wild-type (WT) 3’-UTR or mutant (MUT) 3’-UTR of NONMMUT055714 and SORLA were amplified and subcloned into psiCHECK-2 luciferase reporter vector, respectively. HEK-293 cells were first transfected with plasmids for 48 hours. Cells were then washed 3 times with PBS and cell lysis buffer was then added. The lysates were transferred to a 96-well luciferase activity detection plate. Luciferase Assay Reagent (Meilunbio, Dalian, China) was added to each well, and the activity of firefly luciferase was detected immediately after mixing. Stop and Glo Reagent (Meilunbio, Dalian, China) was quickly added to each well, and renilla luciferase activity was detected immediately after mixing. The relative activity of luciferase was determined by the ratio of activity between renilla luciferase and firefly luciferase.

### qRT-PCR

Total cell RNA was extracted from tissues as directed by the Trizol kit (Tiangen, Beijing, China), and cDNA was synthesized by reverse transcription according to protocol of the Trans Script First-Strand cDNA Synthesis Super Mix (Tiangen, Beijing, China). The RT-PCR reaction was performed according to protocol of the Trans StartGreen q PCR Super Mix kit (Tiangen, Beijing, China). Primers were designed by Primer 5.0 software and synthesized by Beijing Aoko Biological Company. The PCR reaction conditions were as follows: denaturation at 95° C for 5 min, followed by 45 cycles of 95° C for 15s, 60° C for 15s and 72° C for 15s. The amplified products were detected by 1% agarose gel electrophoresis, and Quantity One software was used to analyze the band intensity and internal parameters.

### Western blotting

Dissected hippocampal tissues or cell samples were added to lysis buffer (Solarbio, Beijing, China), then ultrasonicated and centrifuged at 13,000 rpm for 10 min. The supernatant was collected and protein quantification was conducted via the Lowery method. Fifty micrograms of total proteins were added to a 10% SDS-PAGE gel (Solarbio, Beijing, China), separated by electrophoresis, and transferred to polyvinylidene fluoride membrane, which was blocked with 5% skim milk (Solarbio, Beijing, China). Membranes were incubated with primary antibodies SORLA (ab190684; Abcam, Cambridge, UK), Aβ (ab2539; Abcam, Cambridge, UK), Phospho-Tau (#20194; Cell Signaling Technology, USA), and GAPDH (#5174; Cell Signaling Technology, USA) were incubated overnight at 4° C. Secondary antibody (#7074; Cell Signaling Technology, USA) were added, and the ECL method was used to measure luminescence. X-ray scanning was used for imaging, and Seion-Image software was used for quantitative analysis of the results.

### Flow cytometry

To examine the effect of NONMMUT055714 expression on cell apoptosis, we performed flow cytometry on each group of primary hippocampal neurons. Cells were collected, washed with PBS buffer (Solarbio, Beijing, China), and digested with trypsin (Solarbio, Beijing, China). Cell density was adjusted to 5 × 10^5^/ml. The cells were suspended with 500 μl binding buffer, mixed with 10 μl Annexin V-FITC and 10 μl PI (C1062M; Beyotime Biotechnology, Shanghai, China), then incubated and stained at room temperature in the dark for 15 min. A FACS flow cytometer was used to quantify cellular fluorescence and observe the percentage of apoptotic cells.

### ELISA assay

Hippocampal neurons were isolated during logarithmic growth, and the number of neurons was adjusted to 1×10^5^/ml before addition to a 12-well plate at 1.0 ml/well. The supernatant was collected after cells were incubated at 5% CO_2_ at 37° C for 48 h. The concentrations of IL-1, IL-6, and TNF-α in the culture supernatant were measured according to the instructions of the ELISA kit (Abcam, Cambridge, UK).

### Detection of oxidative stress

Colorimetry was used to detect changes in malondialdehyde (MDA), a product of chemical reactions that take place during oxidative stress. The level of 8-iso-Prostaglandin F2α (8-iso-PGF2α) were measured by ELISA assay (Mouse 8-iso-PGF2α(8-isoprostane) ELISA Kit, EM1579, Wuhan Fine Biotech, China). The catalase (CAT) content was determined by spectrophotometry.

### Statistical analysis

No sample size calculations were performed. Sample sizes were determined based on prior studies using similar experimental paradigms. Data are presented as the mean ± Standard Deviation (SD) from at least three independent experiments. Unpaired *t*-tests were used to analyze the difference between two groups, and one-way analysis of variance (ANOVA) was used for analyzing the differences between multiple groups. Differences with *p* <0.05 were considered statistically significant. SPSS version 20.0 software was used for statistical analysis.

## RESULTS

### NONMMUT055714 is downregulated in the mouse model of POCD and is associated with decreased performance on cognitive testing

**Figure 1:**
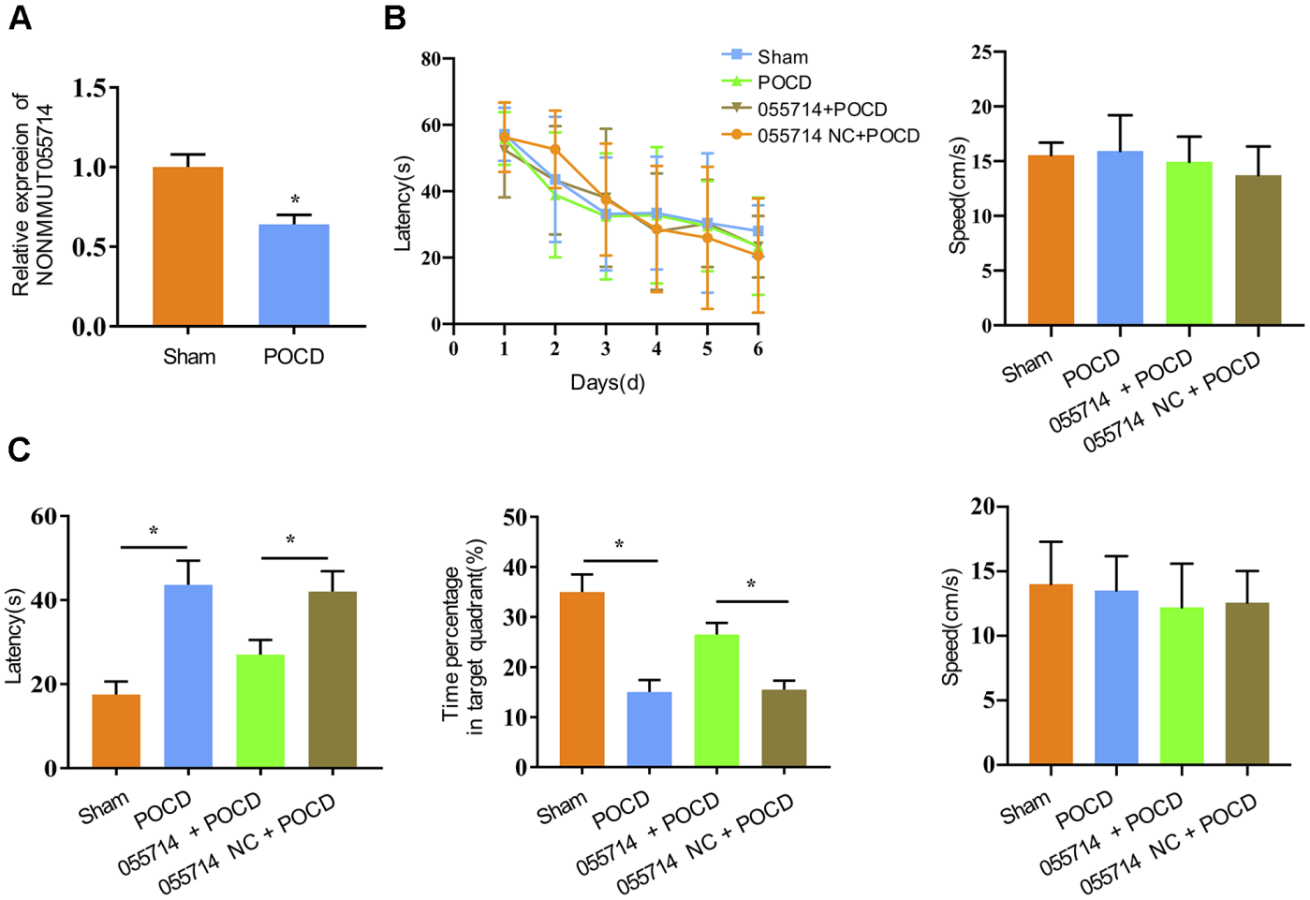
**Overexpression of lncRNA NONMMUT055714 significantly preserves cognitive performance after surgery.** (**A**) The relative mRNA levels of NONMMUT055714 in sham and POCD mice. (**B**) Results of Morris water maze training trials in each group of aged mice. (**C**) Comparison of probe trial performance between groups. N = 8 per group. Data represented as mean ± SD; * indicates *p* < 0.05.

Previous microarray analysis performed by our group identified differential expression of lncRNAs in the hippocampus of POCD and control mice [17]. In this study, the expression of NONMMUT055714 was significantly downregulated in POCD mice compared to the sham group (p < 0.01) (Figure 1A). To explore the potential role of NONMMUT055714, we carried out the Morris water maze to assess spatial learning and memory ability [21]. With additional days of training, latency for the mice to identify the underwater platform decreased in all groups, while there was no difference in escape latency and swimming speed among groups (Figure 1B). When the POCD mice underwent the probe trial on the 3rd day after surgery, their latency to reaching the platform was significantly prolonged compared with that of the sham group (sham vs. POCD, mean difference [MD] 26.28 seconds, 95% confidence interval [CI] 21.19 to 31.36, p < 0.05). POCD mice also spent a lower percentage of time in the target quadrant (sham vs POCD, MD -19.88%, 95% CI -23.16 to -16.60, p < 0.05) (Figure 1C). Overexpression of NONMMUT055714 significantly reduced escape latency (055714 + POCD vs. 005514 NC + POCD, MD 11.53 seconds, 95% CI 6.98 to 16.06, p < 0.05) and increased the time percentage in target quadrant (055714 + POCD vs. 005514 NC + POCD, MD -11.02, 95% CI -13.29 to -8.74, p < 0.05) in POCD mice. There was no difference in swimming speed between any groups during the probe trail test (Figure 1C). These findings suggest that overexpression of NONMMUT055714 protects against the cognitive impairment of POCD mice.

### Silencing NONMMUT055714 induces key biochemical features of POCD *in vitro*

**Figure 2:**
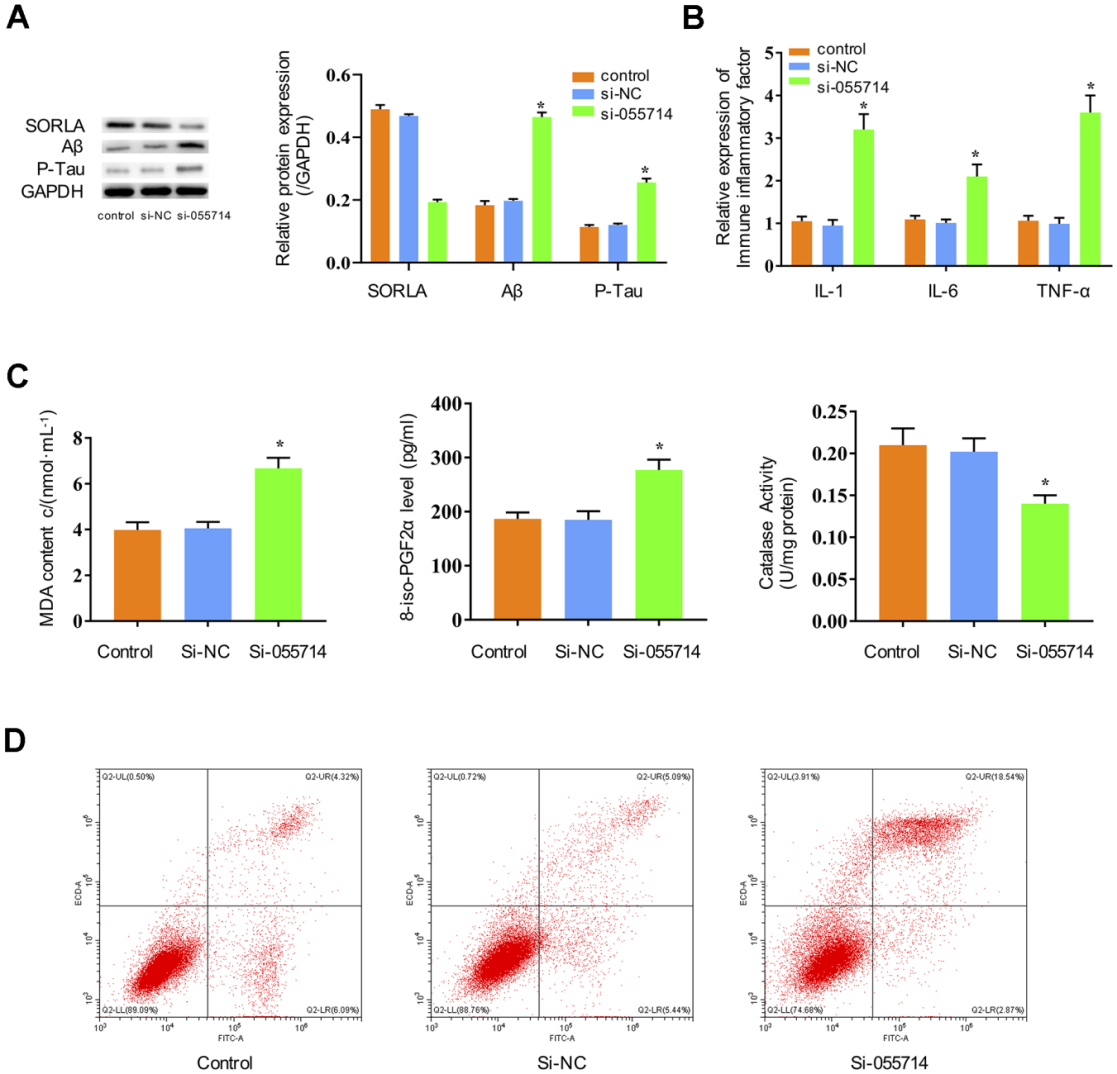
**Influence of NONMMUT055714 expression on inflammatory markers, oxidative stress, tau protein phosphorylation, and neuronal apoptosis in primary hippocampal neurons.** (**A**) Differential expression of SORLA, Aβ and P-Tau by Western Blot. (**B**) Expression of IL-1, IL-6, and TNF-α. (**C**) Quantitative analyses of MDA, 8-iso-PGF2α, and CAT levels in neurons. (**D**) Percentage of hippocampal neuron apoptosis by flow cytometry. N = 3 from three independent experiments. Data represented as mean ± SD; * indicates *p* < 0.05.

To study the potential functions of NONMMUT055714, we investigated whether its expression impacts inflammatory responses, oxidative stress, and tau phosphorylation in primary hippocampal neurons. Suppression of NONMMUT055714 significantly decreased the expression of SORLA in the si-NONMMUT055714 group of primary hippocampal neurons compared to the control and si-NC groups (*p* < 0.05) (Figure 2A). The si-055714 group demonstrated significantly increased expression of Aβ and phosphorylated tau protein (Figure 2A) as well as inflammatory markers IL-1, IL-6, and TNF-α (*p* < 0.05) (Figure 2B). Compared to the control and si-NC groups, the si-055714 group had significantly increased levels of MDA and 8-iso-PGF2α, and a significantly decreased level of CAT (*p* < 0.05) (Figure 2C). Increased apoptosis was also observed on flow cytometry in si-055714 group relative to the NC group (*p* < 0.05) (Figure 2D). Taken together, these results indicate that silencing NONMMUT055714 promotes inflammatory responses, oxidative stress, neuronal apoptosis and tau phosphorylation in primary hippocampal neurons.

### NONMMUT055714 binds miR-7684-5p to potentially influence SORLA expression

**Figure 3:**
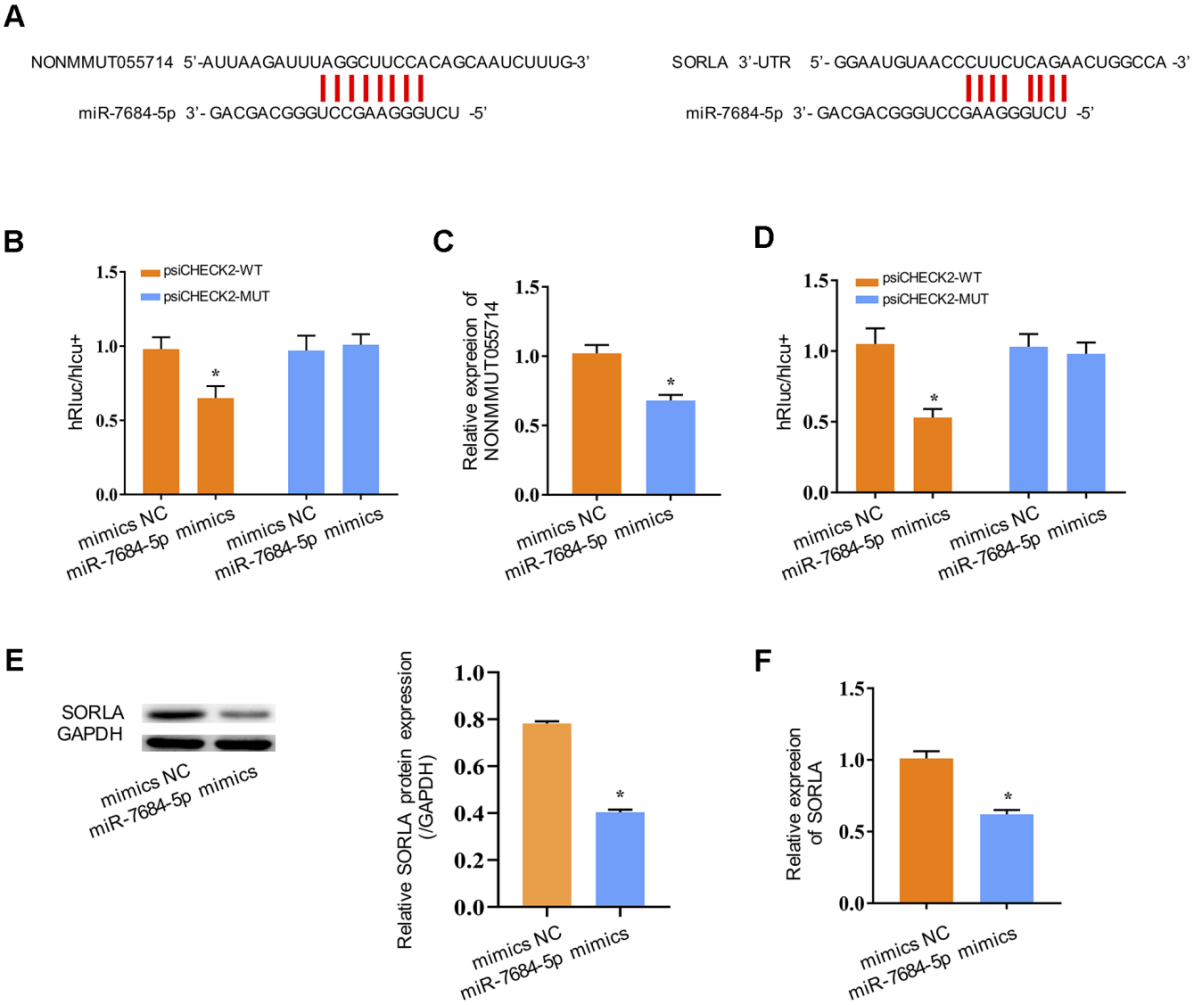
**NONMMUT055714 and SORLA as binders of miR-7684-5p.** (**A**) Predicted binding sites of miR-7684-5p to NONMMUT055714 and SORLA. (**B**) Dual-luciferase reporter experiments indicating NONMMUT055714 as a binder of miR-7684-5p. (**C**) The relative mRNA levels of NONMMUT055714 in the setting of miR-7684-5p overexpression. (**D**) SORLA as a potential target of miR-7684-5p. (**E**) Levels of SORLA protein in primary hippocampal nerve cells in the setting of miR-7684-5p overexpression. (**F**) Levels of SORLA mRNA in primary hippocampal nerve cells in the setting of miR-7684-5p overexpression. N = 3 from three independent experiment. Data represented as mean ± SD; * indicates *p* < 0.05.

We next explored the molecular mechanism underlying the function of NONMMUT055714. Many lncRNAs are known to function as a ceRNA to modulate the expression and biological functions of miRNA. The bioinformatics databases MiRanda and PicTar identified one potential binding site on miR-7684-5p for NONMMUT055714 (Figure 3A). The TargetScan bioinformatics algorithm identified one potential binding site on SORLA for miR-7684-5p (Figure 3A). To further explore the underlying mechanism of the lncRNA/miRNA regulatory function, dual-luciferase reporter assay was performed. MiR-7684-5p mimics reduced the luciferase activity of wild-type NONMMUT055714 reporter vector psiCHECK2-WT (*p* < 0.05), but not that of mutant reporter vector psiCHECK2-MUT (Figure 3B). MiR-7684-5p overexpression was associated with a significant decrease in NONMMUT055714 expression (*p* < 0.05) (Figure 3C), indicating that there exists mutual regulation between miR-7684-5p and NONMMUT055714. The miR-7684-5p mimics also led to the attenuation of fluorescence of the wild-type 3’-untranslated region (WT 3’-UTR) in SORLA (*p* < 0.05), but had no effect on SORLA with mutant (MUT) 3’-UTR (Figure 3D). Expression of SORLA protein and SORLA mRNA were significantly reduced in primary hippocampal neurons with miR-7684-5p overexpression (*p* < 0.05) (Figure 3E, 3F). These results suggest that SORLA is a direct target of miR-7684-5p.

### Downregulation of miR-7684-5p rescues the role of NONMMUT055714 *in vitro*

**Figure 4:**
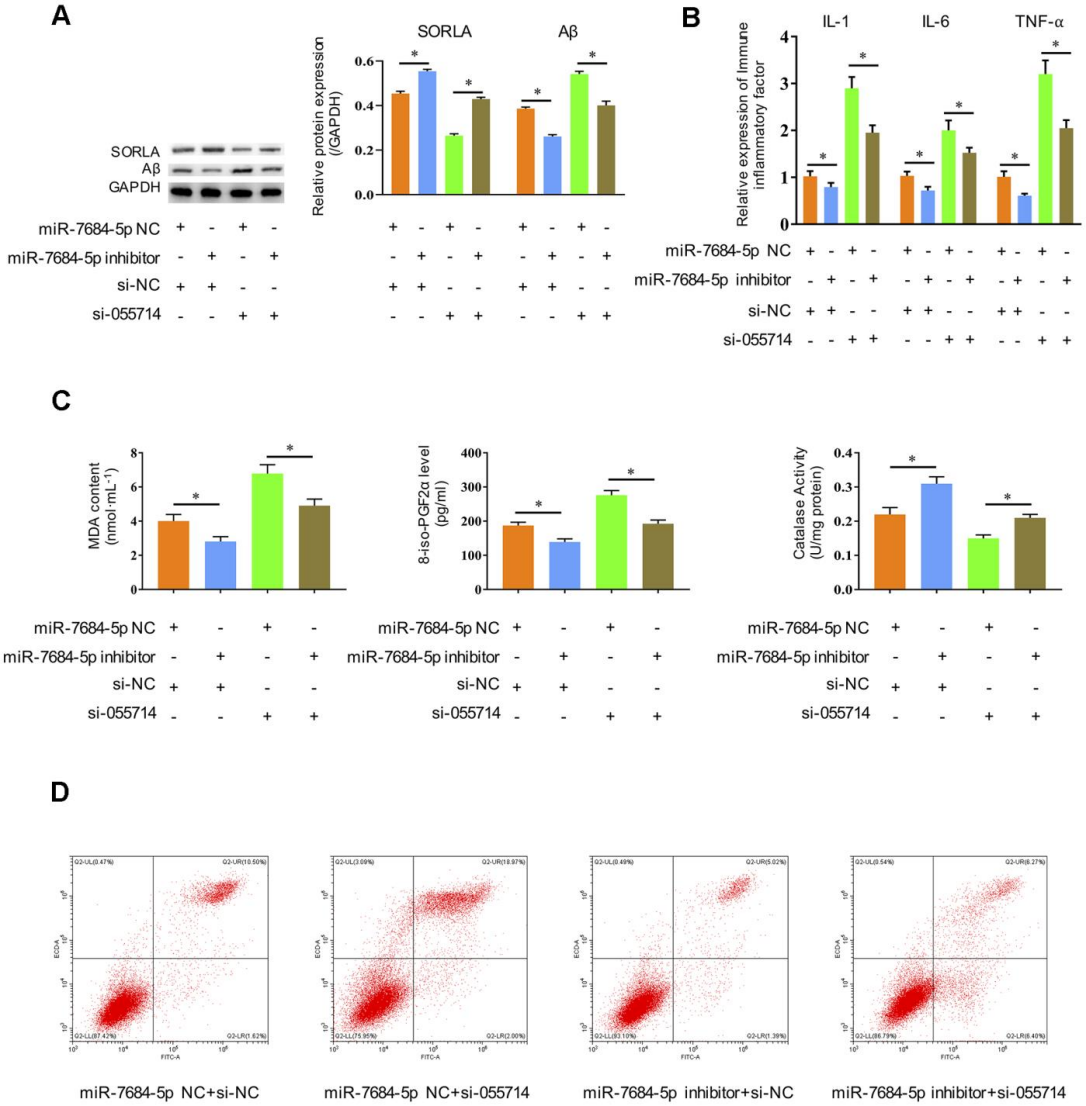
**Silencing miR-7684-5p restores the effects of silencing NONMMUT055714 in primary hippocampal neurons *in vitro*.** (**A**) The protein expression of SORLA, Aβ and P-Tau by western blot. (**B**) The expression of IL-1, IL-6, and TNF-α, as determined by ELISA assay. (**C**) Silencing miR-7684-5p reverses the effect of si-NONMMUT055714 on markers of oxidative stress. (**D**) Percentage of hippocampal neuron apoptosis by flow cytometry. N = 3 from three independent experiment. Data represented as mean ± SD; * indicates *p* < 0.05.

We then investigated the influence of miR-7684-5p downregulation on the role of NONMMUT055714 in POCD. Significantly decreased SORLA expression and increased Aβ expression were observed with silencing of NONMMUT055714 in transfected primary hippocampal neurons *in vitro*. Silencing of miR-7684-5p reversed these effects, with SORLA and Aβ expression similar to negative controls (*p* < 0.05) (Figure 4A). The expression of IL-1, IL-6, and TNF-α were significantly increased with si-NONMMUT055714 transfection, while there was a relative decrease in these markers with the addition of miR-7684-5p inhibitor transfection (*p* < 0.05) (Figure 4B). Silencing of miR-7684-5p also reversed si-NONMMUT055714-induced increases in MDA and 8-iso-PGF2α and decrease of CAT (*p* < 0.05) (Figure 4C). In flow cytometry, silencing of miR-7684-5p eliminated the effect of si-NONMMUT055714 increasing the rate of apoptosis, with the percentage of apoptotic neurons similar to the negative control group (*p* < 0.05) (Figure 4D).

### NONMMUT055714 regulates SORLA and Aβ expression through miR-7684-5p *in vivo*

**Figure 5:**
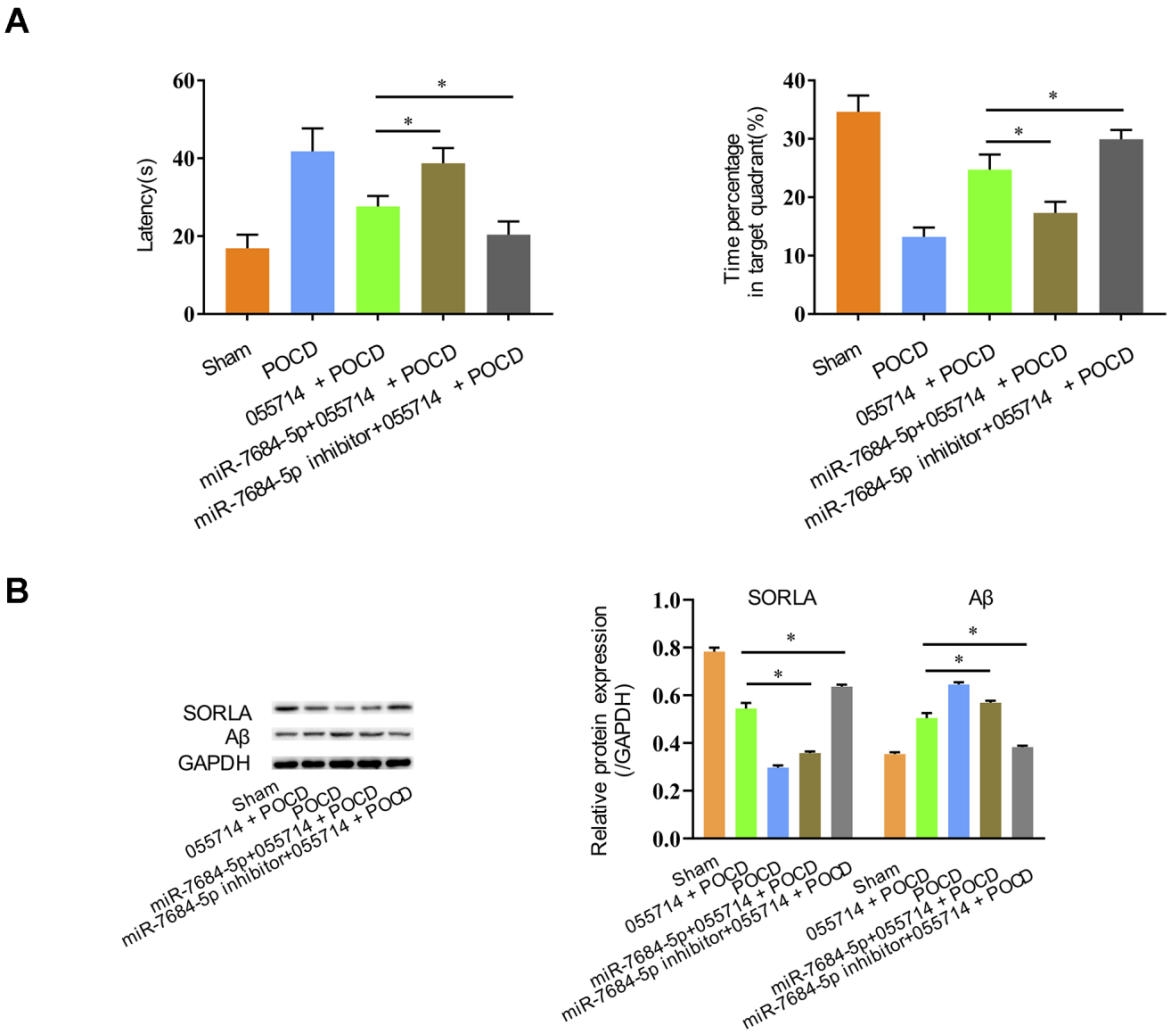
**Silencing miR-7684-5p uncovers the effect of NONMMUT055714 on postoperative cognitive function in aged mice.** (**A**) Latency time and percentage time spent in target quadrant in the probe trial. (**B**) Differential expression of SORLA and Aβ by Western Blot. N = 8 per group. Data represented as mean ± SD; * indicates *p* < 0.05.

We further evaluated the role of NONMMUT055714 in POCD through miR-7684-5p expression. Overexpression of NONMMUT055714 improved cognitive performance in POCD mice, but overexpression of miR-7684-5p reversed those effects, with prolonged latency (055714 + POCD vs. miR-7684-5p + 055714 + POCD, MD 11.15 seconds, 95% CI 7.53 to 14.78, *p* < 0.05) and decreased time percentage in the target quadrant (055714 + POCD vs. miR-7684-5p +055714 + POCD, MD -7.41, 95% CI 9.89 to -4.92, *p* < 0.05) (Figure 5A). However, miR-7684-5p inhibitor enhanced the protective effect of NONMMUT055714 in POCD, with reduced latency (055714 + POCD vs. miR-7684-5p inhibitor + 055714 + POCD, MD -7.22 seconds, 95% CI -10.55 to -3.88, *p* < 0.05) and a greater percentage of time in the target quadrant (055714 + POCD vs. miR-7684-5p inhibitor + 055714 + POCD, MD -5.20, 95% CI 2.85 to 7.55, *p* < 0.05) (Figure 5A).

Overexpression of miR-7684-5p decreased SORLA expression and increased Aβ expression in miR-7684-5p + 055714 + POCD mice compared to 055714 + POCD mice, but silencing miR-7684-5p reversed that effect caused by lncRNA NONMMUT055714 (*p* < 0.05) (Figure 5B).

## DISCUSSION

Postoperative cognitive dysfunction (POCD) is a neurological complication of surgery especially common in elderly patients [1]. POCD is characterized by changes in orientation, difficulty concentrating, memory loss, and decrease in executive ability [8]. The biological and molecular mechanisms underlying development of POCD are poorly understood. Like other progressive neurological diseases, the complex network of transcriptional regulation and gene expression in the brain is likely to play an important role in POCD pathogenesis [13].

LncRNAs represent an important area of research investigating the pathogenesis of POCD. Several recent studies have identified lncRNAs which are differentially expressed in POCD [22–24]. Among these, Chen et al. demonstrated that knockdown of the lncRNA PCAI decreased cell death and degree of inflammatory response in BV-2 cells treated with lipopolysaccharide *in vitro* [25]. Microarray analysis previously performed by our group demonstrated that the lncRNA NONMMUT055714 is significantly downregulated in the hippocampus of a POCD mouse model compared to control mice [17]. The cause of NONMMUT055714 downregulation in the postoperative setting remains unclear.

In this study, we focused on the role of NONMMUT055714 in the development of POCD via regulation of miR-7684-5p. We confirmed again that NONMMUT055714 has decreased expression in a mouse model of POCD. Lentivirus-induced overexpression of NONMMUT055714 in our POCD model protected against cognitive impairment experienced postoperatively compared to control POCD mice.

We next investigated the effects of NONMMUT055714 on cellular function of hippocampal neurons. Silencing of NONMMUT055714 *in vitro* was associated with decreased SORLA, increased Aβ and p-tau expression, increased inflammatory biomarkers, increased markers of oxidative stress, and increased neuronal apoptosis. Normally, the generation and clearance of Aβ constitute a dynamic balance. When there is impairment of these processes, abnormal and excessive Aβ deposition has been shown to occur, resulting in increased microglial cell activation and inflammatory responses [26], oxidative stress [27, 28], tau protein phosphorylation [29, 30], cell apoptosis [31, 32], and release of pro-inflammatory cytokines including IL-1, IL-6 and TNF-α [33]. Together, these changes characterize the molecular basis and proposed pathogenesis of neurological decline in many neurodegenerative diseases.

We identified miR-7684-5p as a NONMMUT055714-related miRNA, and in turn a potential upstream regulator of SORLA. Sorting-related receptor with A-type repeats, or SORLA, is a 250 kDa transmembrane I protein on chromosome 11 that plays an important role in transporting proteins in neurons [17, 34]. SORLA likely acts as an intracellular sorting receptor for proteins including APP, the precursor to Aβ. Defects in SORLA may contribute to the development of Alzheimer’s disease via its upstream regulation of Aβ production and aggregation [18, 19, 35]. In this study, silencing of NONMMUT055714 reduced expression of SORLA *in vivo* and *in vitro.* Silencing of miR-7684-5p was shown to reverse these effects, again both *in vivo* and *in vitro*. These findings are consistent with previous research from our group showing that miR-7684-5p expression induced hippocampal Aβ accumulation through downregulation of SORLA in a mouse POCD model [36].

In the competing endogenous RNA (ceRNA) hypothesis put forth by Salmena et al., lncRNAs act as ceRNAs, also known as natural miRNA sponges, to competitively bind miRNAs. Together, these interactions form mutually-regulating lncRNA-miRNA-mrRNA networks [15, 16]. We identified NONMMUT055714 as a potential competitive binder of miR-7684-5p. The overexpression of miR-7684-5p resulted in decreased SORLA and increased Aβ expression *in vitro.* In the subsequent cell rescue experiment, silencing of miR-7684-5p reversed the promotion of the inflammatory response, oxidative stress, tau phosphorylation, and cell apoptosis induced by silencing of NONMMUT055714. In the corresponding animal rescue experiment, silencing miR-7684-5p reversed the effects of silencing NONMMUT055714, leading to rescue of cognitive function, decreased Aβ expression, and the promotion of SORLA expression.

Our findings suggest that NONMMUT055714 is protective against the development of POCD. We propose that NONMMUT055714 functions as a miRNA sponge to regulate miR-7684-5p and SORLA, thus influencing inflammation, oxidative stress, tau phosphorylation, and cell apoptosis pathways. NONMMUT055714 is a novel target for the investigation and prevention of POCD deserving of future study.
